# Cataluminescence in Er‐Substituted Perovskites

**DOI:** 10.1002/advs.202101764

**Published:** 2021-08-08

**Authors:** Andreas Borgschulte, Olga Sambalova, Emanuel Billeter, Andrea Sterzi, Jana Niggli, Bastian Welte, André Heel, Reto Holzner

**Affiliations:** ^1^ Laboratory for Advanced Analytical Technologies Empa ‐ Swiss Federal Laboratories for Material Science and Technology Überlandstrasse 129 Dübendorf CH‐8600 Switzerland; ^2^ Department of Chemistry University of Zurich Winterthurerstrasse 190 Zürich CH‐8057 Switzerland; ^3^ Institute of Environmental and Process Engineering (UMTEC) OST ‐ Eastern Switzerland University of Applied Sciences Oberseestrasse 10 Rapperswil CH‐8640 Switzerland; ^4^ Econimo‐Drive AG Gewerbestrasse 11 Cham CH‐6330 Switzerland

**Keywords:** cataluminescence, Cu nanoparticles, methanol combustion, perovskites, plasmon spectroscopy

## Abstract

Thermophotovoltaic devices have promising applications for energy conversion. However, current conversion efficiency of chemical energy to light is very low, limited by the competing process of heat dissipation released as black body radiation. From a fundamental point of view, the direct conversion of chemical energy into light without this detour is possible. This so called cataluminescence from methanol combustion over Er‐substituted SrTiO_3_ with high efficiency is demonstrated. The catalytically active quaternary perovskites Er_0.15_La_0.15_Sr_0.55_Ti_0.95_Cu_0.05_O_3 − *δ*_ exsolute and reabsorb metallic Cu particles onto the surface in reducing and oxidizing conditions, respectively. Thus, it is able to manipulate the surface structure and investigate its influence on the catalytic as well as luminescent properties. The fuel to air ratio around the stoichiometry point changes the conditions from reducing to oxidizing and thereby alters the surface properties. This is evidenced by post mortem X‐ray diffraction and X‐ray photoemission as well as operando optical spectroscopy. Cataluminescence takes place under oxidizing conditions (lean fuel to air mixture) on the Er‐perovskite oxide with a strong selective near infrared emission, while reducing conditions stimulate formation of plasmonic Cu‐nanoparticles, which emit black body radiation.

## Graphical abstract

The catalytically active quaternary perovskite Er_0.15_La_0.15_Sr_0.55_Ti_0.95_Cu_0.05_O_3 − *δ*_ converts the chemical energy released during methanol combustion into light at a higher degree than expected from black‐body radiation. The cataluminescent behavior is analyzed by various in situ and post mortem characterization methods showing the exsolution of Cu and formation of plasmonic nanoparticles, and their effect on the cataluminesence of the material.

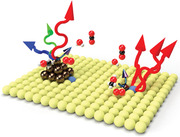

## 1 Introduction

Thermophotovoltaic converters transform chemical energy of basically any fuel to electricity. They are rather simple, and particularly suited to deliver electrical power to remote places for outdoor activities and military applications.^[^ ^1^ ^]^ These devices traditionally consist of a burner, which combusts the fuel at high temperatures to generate light (chemical energy to light conversion), and a photovoltaic cell, which converts the light into electricity. The generated light is mainly black body radiation with main energy distribution in the far infrared region at realistic temperatures. As photovoltaic cells convert light only above the threshold energy, the efficiency of such a thermophotovoltaic device is very low. In the past, selective emitters were developed to increase the efficiency, but with limited success (thermoluminescence).^[^ ^2^ ^]^ Circumventing heat emission (i.e., lower entropy production) may markedly increase the efficiency, extending the applicability of such emitters for energy conversion. Direct conversion of chemical energy to light is known in nature as chemiluminescence. Cataluminescence is a specific type of chemiluminescence that occurs on solid catalysts during heterogeneous fuel oxidation reactions. The phenomenon stems from combustion‐formed radicals present at the surface of the light emitting material (in contrast to the outdated gas lights, where the radicals are formed in the gas phase).^[^ ^3^ ^]^ It is the main non‐thermal factor that determines light emission spectrum, first observed on TiO_2_.^[^ ^4^ ^]^ Since then, the effect was used to develop luminescence sensors for various chemical compounds,^[^ ^5^, ^6^, ^7^ ^]^ which suggests another potential application, as an in situ probe of ongoing catalytic oxidation reactions. Understanding the phenomenon carries tremendous potential from the viewpoint of fundamental science and industry, which can be extrapolated from improving efficiency of methanol combustion to combustion of other fuels.

Thus, the first goal is to develop a material that fulfills the criteria of being catalytically active for methanol oxidation (Equation (1)) while simultaneously possessing electronic states that make use of the released reaction energy of about 1.1 eV by selective emission rather than heat generation.

(1) $${\text{CH}}_{3}\text{OH}+\frac{3}{2}O_{2}\to {\text{CO}}_{2}+2H_{2}O,\;\Delta H=-740\;\text{kJ}\;\text{mol}{}^{-1}$$

Typical methanol oxidation catalysts are based on precious metal nanoparticles deposited on oxide supports.^[^ ^8^, ^9^, ^10^ ^]^ In addition, rare earth oxides from the lanthanide series are well known for their thermoluminescence properties in the near infrared (NIR) region.^[^ ^2^, ^11^ ^]^ For this purpose, we explored the combination of Ru, Pd, and Cu nanoparticles on rear earth oxide supports in preliminary studies. However, we observed no or very inefficient selective emission and the typically low melting points of the precious metal nanoparticles (bulk Cu = 1084 °C) that resulted in a complete loss of catalytic activity already after a short reaction period (< minutes) assigned to sintering of the particles on the surface. Inspired by recent developments of oxide based catalysts,^[^ ^12^, ^13^ ^]^ we propose a different pathway: metal oxide based perovskites with the general formula ABO_3_ are well known for their flexibility in implementing foreign ions into the crystal lattice without major structural reorganization.^[^ ^14^ ^]^ Depending on their ionic radii, dopants can be introduced on the A‐ as well as on the B‐sites leading to a general formula of AA′BB′O_3_. If present, different redox potentials enable exsolution of these dopants as metal nanoparticles on the surface in a reductive atmosphere, with subsequent reabsorption into the crystal lattice in an oxidative atmosphere. This method differs from a simple post synthetic impregnation procedure and generates strong crystal interfaces, as evidenced by, for example, higher thermal stability of the nanoparticles on the surface.

In this paper we demonstrate the cataluminescence in Er‐ and Cu‐ substituted SrTiO_3_ (Er_0.15_La_0.15_Sr_0.55_Ti_0.95_Cu_0.05_O_3 − *δ*_, ErLaSTO). The paper is organized as follows: first section describes the general idea and first outcomes of cataluminescence from methanol combustion. To distinguish the physical effects of black body radiation, we make use of the peculiar properties of the catalytically active quaternary perovskites, which reversibly exsolute and absorb metallic Cu particles onto the surface when switching from oxidizing to reducing conditions.^[^ ^13^ ^]^ We characterize the material using a variety of operando and post‐mortem methods. Particular emphasis is put on the exsolution process, which is followed by in situ plasmon resonance spectroscopy. During combustion, changing fuel to air ratio (“lambda ratio”) around the stoichiometry point from reducing to oxidizing conditions triggers the formation of surface nanoparticles. With this, we are able to link the surface transformations to the emission transition (selective NIR‐emission to black‐body emission) due to the change of the lambda ratio. This is a strong indication that cataluminescence takes place on the Er‐perovskite with a strong selective NIR‐emission, while under reducing conditions black body radiation is emitted from catalytically active Cu nanoparticles.

## 2 Results

**Figure 1:**
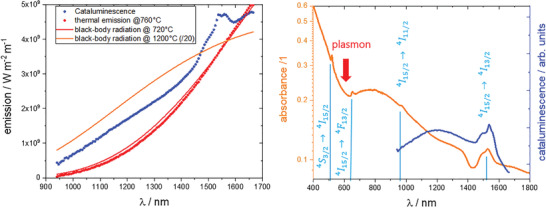
Infrared radiation emitted from a solid catalyzing methanol combustion may originate from black‐body radiation as a result of the heat of combustion and/or from cataluminescence, that is, the transfer of the chemical energy from combustion to luminescent centers. To distinguish the two phenomena, we measure the infrared radiation emitted purely by heating the sample, and during catalytic combustion (see Section 5 and Figure 5 for technical details). Left panel is the NIR‐emission from cataluminescence (blue dots) and thermal emission at 760 °C (red dots) of Er_0.15_La_0.15_Sr_0.55_Ti_0.95_Cu_0.05_O_3 − *δ*_. The red and yellow lines are simulations of black‐body radiation at 720 and 1200 °C, respectively. The right panel compares the black‐body substracted cataluminescence (*T*_BB_ = 1473K) with the corresponding UV–vis–NIR diffuse reflectance spectrum. The broad negative peak around 600 nm is the spectral region of Cu‐plasmon absorption. The sharp peaks are assigned to optical transitions in Er^3 +^ ions in accordance with Er^3 +^ in SrF_2_,^[^ ^15^ ^]^ and Er^3 +^ in sesquioxides.^[^ ^2^, ^16^ ^]^

The catalytically active quaternary perovskite oxide Er_0.15_La_0.15_Sr_0.55_Ti_0.95_Cu_0.05_O_3 − *δ*_ (ErLaSTO) was prepared and characterized as described in Section 5. Briefly, the material is a porous powder consisting of polycrystalline ErLaSTO in the perovskite and double perovskite structure (Figure 6) with a complex surface structure depending on the applied conditions. The powder is put into a modified Harrick cell for catalysis allowing operando UV–vis diffuse reflectance and NIR spectroscopy measurements (see Section 5 for details). The sample can be exposed to flows of different gases, such as air and hydrogen to study the redox behavior, and to air/methanol mixtures for catalytic combustion. The material catalyzes the methanol oxidation reaction (Equation (1)) over the whole experimentally accessible oxygen to methanol ratio *r* = O_2_/CH_3_OH of 1.38 < *r* < 4.6. As the reaction is highly exothermic, it is self‐sustaining after ignition at the temperatures around 400 °C. It follows that the temperature on the catalyst is substantially higher. The infrared radiation emitted from the catalyst is used to estimate its temperature. Neglecting the emissivity, a black‐body model is applied to the NIR spectra. An emission of similar absolute intensity is only obtained if a temperature around 700–800 °C is assumed (see **Figure** **1** and Section 5, Figure 5). However, the NIR emission spectra during catalysis from Er‐substituted perovskite strongly differ from a black‐body spectrum such as observed from the same sample if only heated (blue/red dots in Figure 1). Some features of the cataluminescence spectrum match that of the cathodo/thermo luminescence spectrum of Er_2_O_3_.^[^ ^2^ ^]^ With this, the spectral features can be assigned unambiguously to luminescent emission from Er‐ions which also appear in the diffuse reflectance spectrum (Figure 1).

The selective emission is only present under catalytic conditions. External heating leads to black‐body radiation only. To semi‐quantify the effect, we tuned the emission intensity during catalysis (blue dots in Figure 1) by adjusting the fuel to air ratio in the mixture to the intensity of the thermal emission from the same material exposed to air only at 760 °C, which is the maximum temperature of the sample cell (red dots in Figure 1). The difference between the two measurements gives the efficiency of the emission enhancement in this spectral range by cataluminescence, which is of the order of 70%.

However, the strong enhancement alone is not sufficient enough to prove cataluminescence. The heat release confined to active sites might heat up the surface locally and induce thermoluminescence. The orange curve in Figure 1 is a simulation of black‐body radiation at around 1200 °C divided by a factor of 20, that is, assuming only 5% of the surface is active. The slope of the curve at smaller wavelengths is reproduced, and the selective emission may be an effect from thermoluminescence.^[^ ^2^ ^]^ To counter this argument and to provide evidence of a direct energy transport from the catalytically active site to the luminescent Er‐ions, we make use of the peculiar redox behavior of the material. The B site in ABO_3_ perovskites, here Ti, can be partially substituted by transition metals such as Cu without major changes to the crystal structure as well as electronic properties in oxidizing conditions.^[^ ^13^, ^14^ ^]^ It was found that the material exsolutes metallic Cu particles onto the surface upon reduction at high temperatures. The same behavior follows upon hydrogen exposure at elevated temperature, as confirmed by temperature programmed reduction (TPR), post‐mortem X‐ray diffraction (XRD) and X‐ray photoelectron spectroscopy (XPS) measurements on Er‐substituted perovskites (Section 5).

**Figure 2:**
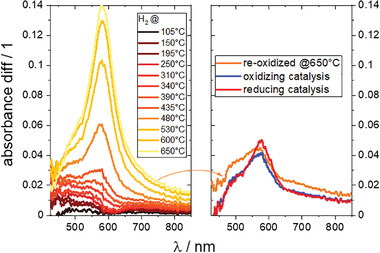
Operando UV–vis diffuse reflectance spectroscopy on Er_0.15_La_0.15_Sr_0.55_Ti_0.95_Cu_0.05_O_3 − *δ*_ during hydrogen exposure from 20 to 650 °C. The right graph shows the subsequently recorded spectra after first exposing to pure air at 650 °C and to methanol / air mixtures at oxidizing (*r* = 2.0) and reducing (*r* = 1.8) conditions. The latter is performed at a cell temperature of 400 °C. The evolution of the peak at 585 nm is indicative of plasmon absorption in Cu‐nanoparticles. The absolute absorbance spectrum of the hydrogenated sample measured ex situ is shown in Figure 1.

The changes observed by XPS post catalysis indicate that also the conditions during catalysis restructure the surface of the Cu‐doped catalysts (Figure 6). However, operando spectroscopy is required, as the surface studied post mortem will not resemble the active surface under catalytic conditions. Optical spectroscopy in the UV–vis range has yielded information on the formation of Cu‐nanoparticles,^[^ ^17^ ^]^ and is rather easily implemented into the experimental setup (see Section 5). The transformation is evidenced by the color change of the samples from yellow to gray upon reduction, indicative of light absorption by metallic nanoparticles. The operando UV–vis diffuse reflectance spectroscopy (**Figure** **2**) shows the evolution of the absorbance peak during heating of an as prepared ErLSTO sample in hydrogen atmosphere. In good agreement with TPR experiments and XPS analysis (see Figure 6), optical changes indicating hydrogen reacting with the material appear already at temperatures as a low as 200 °C. However, the typical peak around 580 nm, indicative of plasmon excitation in Cu nanoparticles,^[^ ^17^, ^18^, ^19^, ^20^ ^]^ develops at higher temperatures. This coincides with the post mortem XRD analysis, detecting Cu‐nanoparticles after hydrogen treatment at *T* > 650°C. The exsolution thus proceeds in two steps: emerging of surface copper atoms at low temperature, which is followed by diffusion and agglomeration to Cu‐nanoparticles at higher temperatures. This process is assumed to be reversible. Exposing the freshly reduced sample to air at 900 K reduces the plasmon peak markedly, but not entirely. In particular, the shoulder at around 500 nm remains, which is assigned to CuO.^[^ ^20^ ^]^ Full reversibility is expected to be reached at higher temperatures than possible in the optical cell.

The optical setup allows the measurement of absorbance changes under catalytic conditions. The intensity of the plasmon excitation depends markedly on the air‐fuel ratio: the peak develops when changing from oxidizing (*r* = 2.0) to reducing (*r* = 1.8) conditions during methanol combustion, without any other spectral changes. This is a strong evidence for the formation/growth of nanoparticles under reducing conditions. We analyzed the products using a gas IR‐spectrometer with particular focus on CO_2_ and CO yields. The CO_2_ yield is directly proportional to the rate of conversion. It is smaller under reducing conditions than under oxidizing conditions, that is, the conversion rate is slower. The CO yield is the opposite. As CO is a strong reducing agent, it can drive the exsolution of Cu and formation of Cu nanoparticles regardless of the overall oxidizing stoichiometry ratio.

**Figure 3:**
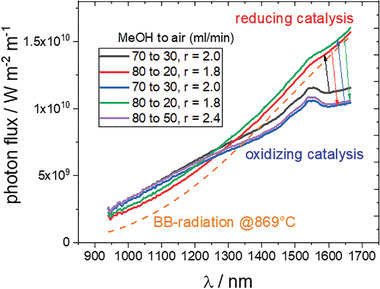
Near infrared emission during catalytic combustion of methanol. The inset gives the flows of methanol‐saturated air to pure air mixture changed in consecutive order (indicated by arrows). Note that the oxygen to methanol ratio, *r*, is decisive for the emission spectrum, while the absolute flows are less relevant.

The structural modifications coincide with the infrared emission, which changes around the ratio 1.8 < *r* < 2.0 from selective emission of Er‐ions to a spectrum similar to black‐body radiation (**Figure** **3**). The effect is reversible and solely dependent on the *r* ratio. The methanol flow, that is, the effective heat release over the catalysts has little effect on the spectral shape as has the exact lambda ratio, as long as *r* does not approach the critical value around 1.9. Cu‐nanoparticles are known to be catalytically active, and are preferential as reaction sites under reducing conditions, as opposed to the sites in the vicinity of the Er‐ions as is the case under oxidizing conditions. The heat evolved is then emitted mainly as black‐body radiation.

## 3 Discussion

The results imply that the radiation released during catalytic combustion is markedly shifted toward more usable short wavelengths by pumping and radiative de‐excitation of Er^3 +^ ions. The question arises about how the energy is transferred from the catalytic centers to the Er‐ions during oxidizing conditions, and how the corresponding electrons are excited. The oxidation of methanol can be described as a series of redox‐processes: six electrons are transferred from methanol to the oxygen atoms bound in CO_2_ and water. Without energy transfer, as is the case on purely metallic catalysts, the corresponding electrons, originally at high chemical potential, dissipate their energy in the metal by a heating process. The same phenomenon was observed on the pre‐experiments, where the combustion took place over catalytically active metal particles on an oxide support. Although the catalysis can be very effective,^[^ ^9^ ^]^ the energy is solely emitted as heat (black‐body radiation, pre‐experiments not shown). The idea of using Cu‐doped perovskites is based on the fact that these materials are catalytically active electrode materials.^[^ ^13^ ^]^ Clearly, the strategy is successful as evidenced by the selective emission from Er‐ions indicating an energy transfer taking place from the catalytically active sites to the luminescent centers. The experimental outcome demonstrates that Cu‐nanoparticles and not Er‐ions are catalytically active. However, although plasmonic Cu nanoparticles have higher catalytic effect than oxidized Cu, they suppress selective emission from Er‐ions (Figure 3). Only if Cu does not quench the excited state, energy transfer takes place. A similar effect had been found on Dy and Cu containing glasses, in which the photoluminescence from Dy ions is quenched once plasmonic Cu nanoparticles are formed.^[^ ^21^ ^]^

The combustion process proceeds via various intermediates, in particular excited radicals.^[^ ^3^, ^4^, ^5^, ^7^ ^]^ On oxides, excitons may be formed by these radicals and thereby stimulate emission as suggested by early works on thoria‐catalyzed CO oxidation.^[^ ^4^ ^]^ However, once the excited state is created, its energy must be transferred to the luminescent Er‐centers to emit selective radiation. Defects in semiconductors are known to suppress luminescence.^[^ ^22^ ^]^ Vacancies may also enhance the overall thermoluminescence,^[^ ^23^ ^]^ however, releasing unselective radiation,^[^ ^24^ ^]^ unless the energy is transferred to luminescent centers.^[^ ^11^, ^25^ ^]^ Following this line of thought, a perfect oxide surface, for example, Er_0.15_La_0.15_Sr_0.55_TiO_3_, without plasmonic Cu nanoparticles and without surface defects should show highest selective cataluminescence. Unfortunately, oxides are seldom catalytically active and if so, it is due to the presence of the defects.^[^ ^26^ ^]^ Neither La_0.3_Sr_0.55_TiO_3_ nor the Er‐substituted material show catalytic activity, and accordingly exhibit no significant emission. Catalytically active sites are thus crucial. As such, Cu nanoparticles were shown to enhance photoluminescence as long as they do not exhibit plasmonic behavior,^[^ ^21^ ^]^ while enabling catalysis in Er‐substituted perovskites.

**Figure 4:**
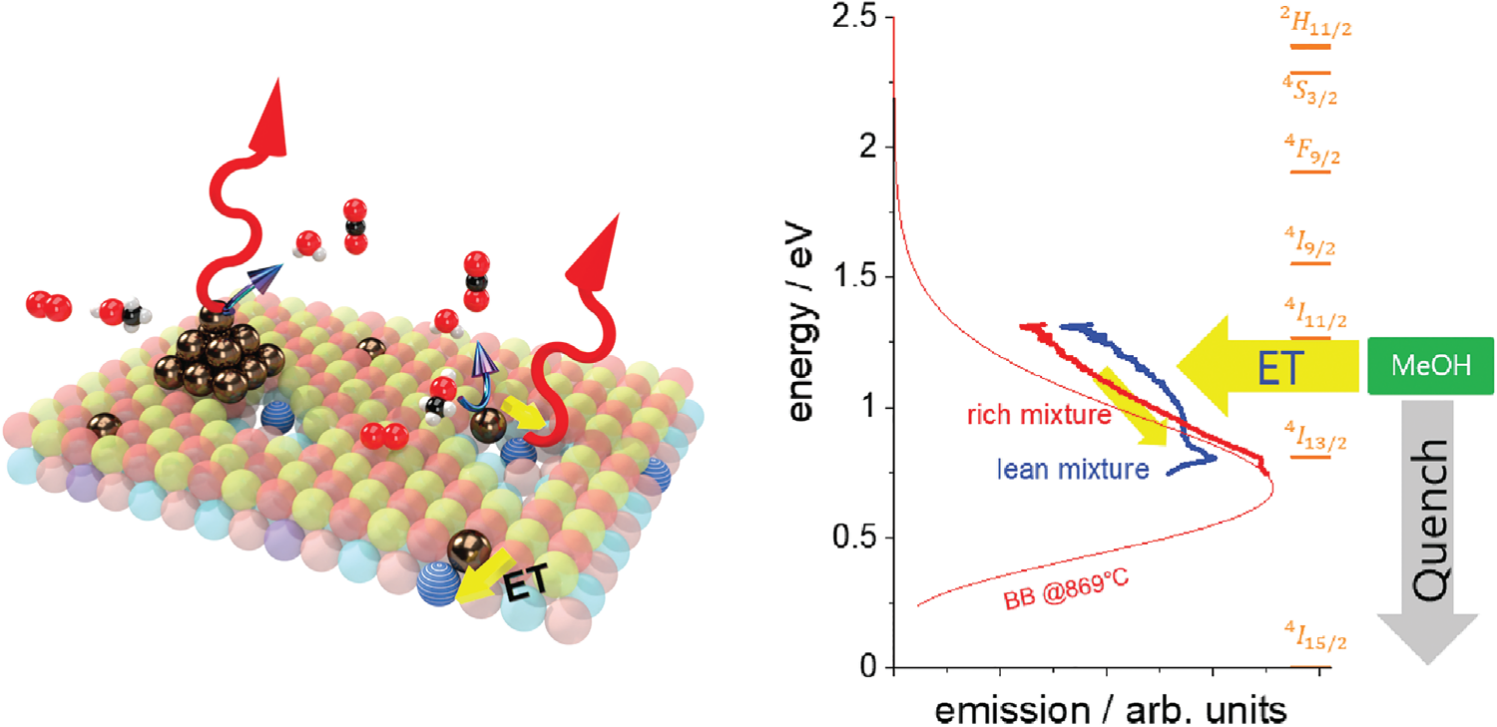
Left panel: Simplified scheme of cataluminescence from methanol combustion on Er_0.15_5La_0.15_Sr_0.55_Ti_0.95_Cu_0.05_O_3 − *δ*_, in lean fuel‐air mixtures (oxidizing conditions), and in rich fuel‐air mixtures (reducing conditions). The right figure compares the corresponding NIR and black‐body spectra, alongside possible transitions in Er^3 +^ ions. ET denotes energy transfer from the catalytically active sites (Cu, golden spheres embedded in the perovskites) to the Er ions (blue). Plasmonic Cu nanoparticles (pyramide made of golden spheres) quench the luminescence.

We summarize the proposed excitation pathway of cataluminescence in **Figure** **4**. Er^3 +^‐ions are pumped either directly or via the formation of excitons by non‐plasmonic, but catalytically active Cu. The origin of the selective emission is the radiative de‐excitation of these Er^3 +^ ions.^[^ ^2^ ^]^ The averaged chemical potential difference per electron gained from methanol combustion is around 1.2 eV, which is just sufficient to pump the ^4^ *I*_13/2_ states. The nature of the pumping (energy transfer, ET) cannot be clarified in this paper. It is well known that the thermoluminescent efficiency depends very sensitively on the absolute position of the 4f electron energy levels with respect to the conduction and to the valence bands.^[^ ^11^ ^]^ This effect may be combined with the exciton formation and emission as proposed for the non‐selective cataluminescence mechanism.^[^ ^4^ ^]^ That is, the excited radicals formed on catalytically active but non‐plasmonic Cu create excitons, whose energy is transferred to the Er‐ions. It is not expected that the energy transfer is perfect, that is, the catalytically active sites will heat up in any case. The observed temperature increase is then an unwanted side effect. Under reducing conditions, the copper particles become plasmonic and quench the hot electrons resulting in heat emission, that is, black body radiation with maximum at lower energies (Figure 4).

Further mechanisms elucidation may be performed by following the relaxation of the excited states over time with ultra‐fast NIR spectroscopy. Alternatively, the effect dependence on concentration of Cu, and rare‐earth ions in the perovskite can be investigated, which is a work in progress. The quenching of the chemically excited states may be further hindered by sample geometry and heat control. The experiments at hand gave indication of this. However, powder samples are suboptimal for this and need to be replaced by defined thin films.

## 4 Conclusions

Aim of this study was to alter the optical properties of a catalyst to suppress the black‐body radiation and generate NIR in a selected energy range during catalytic methanol combustion. We demonstrated the selective IR‐emission from Er‐substituted SrTiO_3_ during catalysis. Careful post‐mortem as well as operando characterizations of quaternary perovskite Er_0.15_La_0.15_Sr_0.55_Ti_0.95_Cu_0.05_O_3 − *δ*_ brought to fore the microstructural changes upon reaction conditions. The results provide arguments for cataluminescence, that is, non‐thermal chemical energy conversion to light. The fraction of light with an energy above 0.8 eV generated by cataluminescence is markedly higher than obtained by black‐body radiation making it attractive for future energy conversion devices. However, the selective NIR‐emission lies in the range suboptimal for adjacent conversion to electricity in Si‐based photovoltaic devices. Further work will thus be needed to utilize rare‐earth ions with a better suited emission energy, and/or upconversion to the desired wavelengths. Research is needed to elucidate the role of Cu‐nanoparticles, and to optimize their impact.

## 5 Experimental Section

### Optical Cell for Emission Measurements and Operando UV–Vis Diffusive Reflectance Spectroscopy

**Figure 5:**
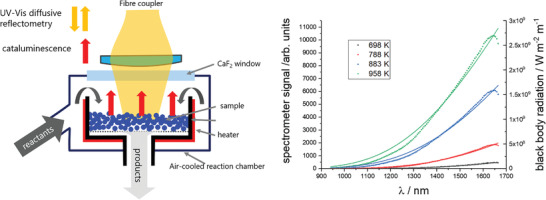
Left: Sketch of the optical cell used both for measuring the emission from the sample during catalysis and UV–vis diffusive difference spectra. For cataluminescence measurements, an NIR‐spectrometer is attached to the fiber coupler (NIR light indicated by red arrows); for UV–vis diffuse reflectance spectroscopy, a split fiber is attached through which UV–vis light is brought to the sample, and the reflected part measured by a UV–vis spectrometer (light in and out indicated by yellow arrows). The right panel shows the calibration measurement making use of the black‐body radiation from a similar but not luminescent sample La_0.3_Sr_0.55_Ti_0.95_Cu_0.05_O_3 − *δ*_.

The aim of this study is to provide a quantitative estimate of the selective light emission from a catalyst during catalytic combustion (cataluminescence). For this a setup was developed, in which the catalyst was exposed to methanol air mixture, while the emitted NIR radiation was measured through a CaF_2_ window. **Figure** **5** shows the employed optical setup consisting of a modified DRIFTS cell (Harrick cell HVC‐DRP‐3). The sample (powder) was placed over a Ni mesh in a heated metal cell to allow the flow of reactants and products through it. A methanol in air feed (0.5–2.0% vol.) was prepared by mixing two air streams, one of which was passed through a methanol saturator (23 °C corresponding to a methanol vapor pressure of 13 mbar). The two flow streams were independently regulated by manual flow controllers. Typical flows were 70 mL min^−1^ methanol/air plus 30 mL min^−1^ air for oxidizing conditions with a sample weight of ≈50 mg. The light emitted from the sample due to cataluminescence or thermoluminescence was collected by a fiber coupler (Thorlabs HCA 3) and coupled into a low OH fiber (Ocean Insight QP400‐2‐VIS‐BX, transmitting best from 400–2100 nm) to an infrared (NIR) spectrometer (Ocean Optics FLAME‐NIR‐INTSMA25, grating N33: 150 lines per mm) for emission measurements. For UV–vis diffuse reflectance measurements, the sample was illuminated through the same fiber coupler using a split fiber optic cable (Ocean Optics, CUSTOM‐BIFI15765). The light source was a halogen lamp with fiber output, the spectrometer a UV–vis fiber spectrometer (Ocean Optics HR4000).

For calibration of the IR‐emission signal, the cell was filled with a La‐ substituted SrTiO_3 − *δ*_ with microstructural and optical properties similar to the Er‐pervoskites. The cell was then heated to various temperatures at low air flows (10 mL min^−1^) to reduce the temperature gradient in the cell. The measured spectrometer signal was then compared to the expected black body radiation at the corresponding temperatures. One proportionality factor (scaling factor) was sufficient to match measured spectra and black‐body model (Figure 5). Using this factor, a universal calibration curve was derived correcting the small deviations from a non‐ideal transmission function of the fiber and spectrometer. The thus derived luminescence (both thermal as well as cataluminescence) signal *I*_lum_ was measured at various methanol to air mixtures.

Operando UV–vis diffusive reflectance spectroscopy were limited to the measurement of semiquantitative absorbance difference spectra Δ*A*_UV –vis_ with

(2) $$\Delta A_{\text{UV-vis}}=-\ln\frac{I_{\text{UV-vis}}\left(f_{o}\right)-I_{\text{lum}}\left(f_{o}\right)-\left(I_{\text{UV-vis}}\left(f_{r}\right)-I_{\text{lum}}\left(f_{o}\right)\right)}{I_{\text{UV-vis}}\left(f_{o}\right)-I_{\text{lum}}\left(f_{o}\right)+\left(I_{\text{UV-vis}}\left(f_{r}\right)-I_{\text{lum}}\left(f_{o}\right)\right)}$$

The cataluminescence signal, *I*_lum_, was subtracted as it also changes in the UV–vis spectral range when going from oxidizing to reducing flow mixtures (*f*_o_, *f*_r_).

For product gas analysis, the exhaust of the Harrick cell was attached to infrared spectrometer with a 6 cm gas cell (Agilent 600 Series FTIR, Cary 600).

Post mortem UV–vis NIR diffuse reflectance spectra of the powders were measured with Jasco V770 spectrophotometer using powder holder (PSH‐002, Jasco) in the back of the integrating sphere ISN‐923 (Jasco, Easton, MD, USA). A reflectance measurement adapter was applied during the measurement to remove the specularly reflected light component. The reference of BaSO_4_ powder used as an etalon white standard for a baseline correction and acetylene carbon black was used for a dark signal correction.

### Materials Preparation and Characterization

**Figure 6:**
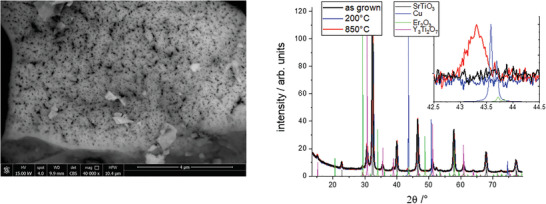
Left panel: SEM micrograph (15 keV acceleration voltage, backscattered electron detector) of the calcinated powder showing the typical porous structure as result of the citrate preparation method. Right panel: XRD analysis of as prepared ErLaSTO (after calcination) and after hydrogen reduction at 200 and 850 °C. All peaks can be assigned to the four phases added from the ICSD reference. The oxide phases hardly change upon reduction, in contrast to the Cu phase, which is clearly visible only after reduction at higher temperatures (inset).

La_0.15_Er_0.15_Sr_0.55_Ti_0.95_Cu_0.05_O_3 − *δ*_ (ErLaSTO) samples were prepared via an in‐house adapted citrate‐gel method^[^ ^12^ ^]^: lanthanum(III) nitrate hexahydrate (99%, abcr GmbH), erbium(III) nitrate pentahydrate (99.9%, Acros organics), strontium(II) nitrate (99%, Acros organics), copper(II) nitrate trihydrate (98%, Carl Roth), and titanium diisopropoxide bis‐acetylacetonate (Puriss, TBAD, Mateck, Germany) were used as starting precursors. Stoichiometric amounts of the metal salts were dissolved in an aqueous solution of citric acid (CA, 99.9%, VWR) and ethylene diamine tetra‐acetic acid (EDTA, 99%, Carl Roth). An ammonia solution (25%, Merck) was used as a pH controlling agent. The ratio of metal ions to CA to EDTA was 1 : 1.5 : 1. After stirring, the precursor solution was placed in a drying furnace for 12 h at 140 °C. The resulting foam was crushed and heat treated for 6 h at 750 °C. **Figure** **6** is a micrograph showing the typical porous structure as a result of the citrate preparation method. The scanning electron microscope (FEI NanoSEM 230) equipped with an EDX‐detector was used to reveal the elemental composition of 68.2 at% O, 15.3 at% Ti, 0.8 at% Cu, 9.3 at% Sr, 3.6 at% La, and 2.7 at% Er. This was very close to the targeted composition given the uncertainty of the EDX analysis.

**Figure 7:**
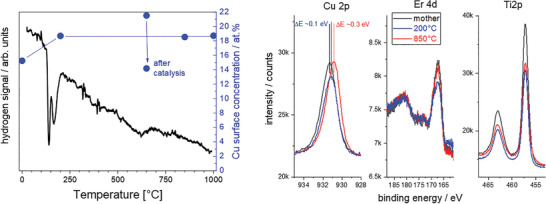
Left panel: Hydrogen signal indicative of the hydrogen consumption by the ErLaSTO during heating in Ar/H_2_ mixture (TPR). The negative peaks mark the reduction process which is associated with the exsolution of Cu as shown by the increase of the Cu surface concentration (blue dots) derived from XPS measurements. Right panel: Selected XPS peaks (Cu 2p, Er 4d, and Ti 2p) of pristine, and reduced samples. The increase of the Cu surface concentration as deduced from the relative intensity ratios is reflected by a small binding energy shift of the Cu 2p peaks.

For phase identification, XRD was measured ex situ on a Bruker D2 Phaser diffractometer with Cu K*α* radiation. For ErLaSTO, XRD proved the formation of the strontium titanate phase (Pm$\bar{3}$m, Figure 6). In addition to this phase, small amounts of the double perovskite phase Y_2_Ti_2_O_7_ were formed. Particularly, no Er_2_O_3_ was formed. Extensive investigation of the hydrogenation process was performed. Temperature programmed reduction (Micromeritics 3Flex) showed a marked hydrogen consumption around 200 °C indicating the reduction of the ErLaSTO (**Figure** **7**), which was usually linked to the exsolution of Cu.^[^ ^14^ ^]^ However, XRD taken after hydrogen treatment at 200 °C did not indicate formation of copper nanoparticles. Peaks associated with these were observed only after reaching reduction temperatures of over 650 °C (Figure 6).

To elucidate the exsolution process, X‐ray photoemission (XPS), and hard X‐ray photoemission spectroscopy (HAXPES) were measured ex situ under ultra‐high vacuum at room temperature on a PHI Quantes spectrometer (ULVAC‐PHI) equipped with an Al and Cr monochromatic X‐ray source for XPS and HAXPES, respectively. The data was analyzed by the CasaXPS software with the calibration based on the O1s peak from lattice oxygen (528.5 eV).^[^ ^27^ ^]^ The measurements of samples as prepared in oxygen atmosphere showed a strong increase of the Cu 2p signal associated with a shift toward lower binding energy after hydrogen treatment at 200 °C (Figure 7). The Cu surface concentration remained roughly constant even after reduction at higher temperatures, indicating that the exsolution process indeed took place at around 200 °C, and higher temperatures promoted the agglomeration of small nanoparticles invisible to XRD to larger particles. As the samples were in contact with air, the metallic Cu nanoparticles were covered with an oxide skin resulting in a binding energy shift of 0.4 eV lower than expected from literature (Δ*E*(Cu‐CuO) = 1.4 eV, Δ*E*(Cu‐Cu_2_O) = 0.1 eV, see Figure 7).^[^ ^28^ ^]^

## Conflict of Interest

The authors declare no conflict of interest.

## Data Availability

The data that support the findings of this study are openly available at: http://doi.org/10.5281/zenodo.5055827.
